# Unification of the family of Garrison-Wright's phases

**DOI:** 10.1038/srep05813

**Published:** 2014-07-24

**Authors:** Xiao-Dong Cui, Yujun Zheng

**Affiliations:** 1 School of Physics, Shandong University, Jinan 250100, China

## Abstract

Inspired by Garrison and Wight's seminal work on complex-valued geometric phases, we generalize the concept of Pancharatnam's “in-phase” in interferometry and further develop a theoretical framework for unification of the abelian geometric phases for a biorthogonal quantum system modeled by a parameterized or time-dependent nonhermitian hamiltonian with a finite and nondegenerate instantaneous spectrum, that is, the family of Garrison-Wright's phases, which will no longer be confined in the adiabatic and nonadiabatic cyclic cases. Besides, we employ a typical example, Bethe-Lamb model, to illustrate how to apply our theory to obtain an explicit result for the Garrison-Wright's noncyclic geometric phase, and also to present its potential applications in quantum computation and information.

As an important measurable physical quantity in conventional quantum mechanics, geometric phase^1^,^2^ not only has many theoretical derivatives^3^,^4^,^5^,^6^,^7^,^8^,^9^,^10^,^11^,^12^,^13^,^14^,^15^ but also attracts much attention due to its potential applications in quantum computation^16^,^17^,^18^,^19^,^20^. Geometric phases in dissipative quantum systems were firstly generalized into the complex number field by Garrison and Wright^7^, and subsequently in the last decade geometric phases at exceptional points (EPs) were investigated for its peculiar properties^12^,^21^,^22^,^23^,^24^,^25^,^26^,^27^,^28^,^29^,^30^. Physically, the real part of complex-valued geometric phases is the geometric phase of usual sense while the imaginary part makes possible to investigate the geometric dilation or contraction of the modulo of a wavefunction. It should be noted that Garrison and Wright modelled a dissipative quantum system by a finite-dimensional time-dependent or parameterized nonhermitian hamiltonian operators with a nondegenerate instantaneous spectrum. It turns out that such a constraint on nonhermitian hamiltonian operators can conduce to effective generalization of concepts of geometric phases in conventional quantum mechanics.

In this paper, we restrict our discussion on the class of such constrained nonhermitian hamiltonian operators modelling biorthogonal quantum systems in order that the family of Garrison-Wright's phases can be extended and unified by a universal theory. Based on the features of the state space of a biorthogonal quantum system, we firstly generalize the concept of Pancharatnan's “in-phase” in interferometery and further present a preparatory theorem which paves the way for the subsequent theory and application. Subsequently, we develop a universal theory for the family of Garrison-Wright's phases, where not only the well-known Garrison-Wright's adiabatic and nonadibatic geometric phases can be integrated, but also the new members of the family, the Garrison-Wright's nonbiorthogonal and biorthogonal geometric phases, can be introduced. And further, we employ a typical example, Bethe-Lamb model, to illustrate how to apply our theory to the Garrison-Wright's noncyclic geometric phase and its potential applications in quantum computation and information. The paper is ended up with conclusion.

## The generalization of pancharatnam's “in-phase”

Supposed that a biorthogonal quantum system is modelled by an *N*-dimensional time-dependent nonhermitian hamiltonian *H*(*t*) with a nondegenerate instantaneous spectrum, then there exists a complete biorthonormal set of basis vectors 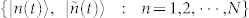 obeying *H*(*t*)|*n*(*t*)〉 = *E_n_*(*t*)|*n*(*t*)〉, 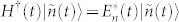, such that^31^

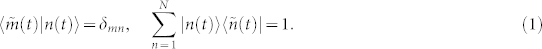
Evidently, a pair of topological vector spaces over complex number field 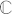, which are in duality, can be defined as 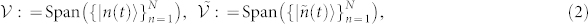
where time parameter *t* in the complete biorthonormal basis implies that this is a moving frame. It should be noted that the topological vector space 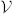 is different from Hilbert space 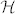 spanned by instantaneous eigenvectors of an *N*-dimensional hermitian *H*(*t*). According to Riesz's lemma, the dual of 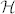 is itself, *i.e.*, 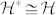, while the dual of 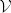 is not itself but 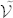, *i.e.*, 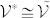.

Also, the fixed rule of state evolution of a biorthogonal quantum system is assumed to be Schrödinger-like equation, 
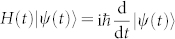
, and whose adjoint, 
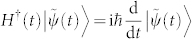
, is introduced for conserving the inner product of evolving states as in conventional quantum mechanics, 
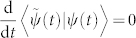
. Without loss of generality, the binormalization of quantum states for nonhermitian quantum system is given by 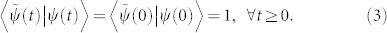
It should be noted that the instantaneous spectrum of *H*(*t*) has no EPs througout the paper. Therefore, it is reasonable to set nonzero initial value of inner product to one. The binormalization Eq.(3) is invariant under local gauge transformation or complex scaling transformation, namely, 
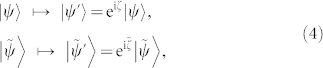
which describes a kind of equivalence relationship. Here 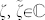 are complex numbers and satisfy 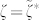. It should be stressed that only the state vector 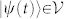 rather than its dual 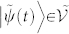 describes a quantum state of the physical system, although they stand equally from their individually satisfying equations.

In conventional quantum mechanics, the interference intensity between any two rays *A*, *B* is written by^6^

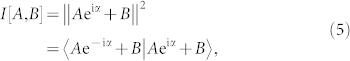
whose superior value, sup*_α_* *I*, implies the stationary condition (namely *δI*/*δα* = 0), and further induces the Pancharatnam's phase *α* = arg〈*A*|*B*〉 which is ill-defined as |*A*〉 is orthogonal to |*B*〉, and the Pancharatnam connection 
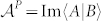
.

Analogously, the generalized interference intensity *J* between any two rays *ψ*_1_, *ψ*_2_ is defined as 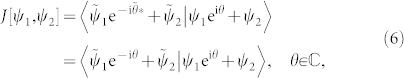
Physically, the definition of the generalized intensity function of interference *J* shows that the complex-valued phase *θ* can be implemented by the *U*(1) phase shift and an amplifier *A* with the range [0, ∞]. As a special case for the definition, quantal interferometry with dissipative internal motion had been considered by Sjöqvist^32^, where *θ* is phase shift acted by the *U*(1) and an absorber *T* with the range [0, 1].

When the generalized interference intensity *J* is stationary with respect to the complex-valued phase *θ*, *i.e.*, *δJ*/*δθ* = 0, the generalized Pancharatnam's phase can be obtained, 
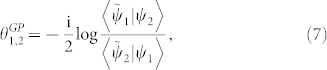
which is ill-defined as 
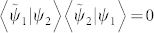
, namely, |*ψ*_1_〉 is biorthogonal to |*ψ*_2_〉.

According to the above discussion, we here address in this paper a central definition which is a generalization for the concept of Pancharatnam's “in-phase” and also the start point of all the subsequent results.

### Definition

*When the generalized interference intensity J[ψ_1_, ψ_2_] is stationary with respect to the complex-valued phase θ such that *
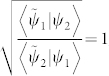
*, then |ψ_1_〉 and |ψ_2_〉 are said to be “in phase” or parallel.*

Actually, the condition 
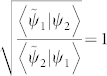
 in the newly generalized definition of “in-phase” equivalently indicates that the phase difference between the two states is zero, which coincides with the condition given by Ref. 6, Im〈*A*|*B*〉 = 0 or arg〈*A*|*B*〉 = 0, which means that |*A*〉 and |*B*〉 are “in-phase”. According to the definition, the global generalized Pancharatnam connection 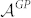 is given by 
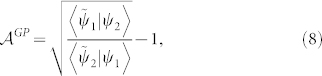
which not only has a physical origin but allow comparing the phase difference between any two neighboring or nonneighboring nonbiorthogonal states.

As the local Pancharatnam connection Im〈*B*(*s*)|*B*(*s* + d*s*)〉 induces a parallel transport law 
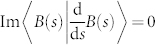
, the local generalized Pancharatnam connection 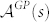


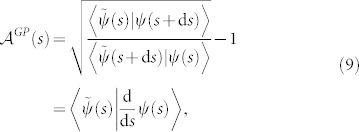
gives a parallel transport law in nonhermitian setting 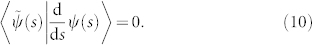
It should be noted that the parallel transport law 
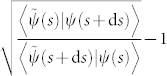
 must be equal to zero, because any nozero complex number can be expressed as an exponential of a complex number which will be taken as the complex-valued phase *θ*, and therefore the “in-phase” condition can not be satisfied. Moreover, the local generalized Pancharatnam connection 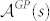 transforms under the gauge transformation Eq. (4) as follow, 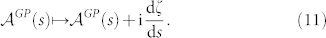
The tangent vector 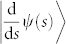 is not gauge covariant, 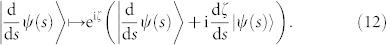
One can check that the covariant derivative 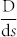 can be defined as 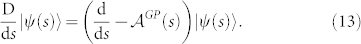
Likewise, the duals of Eqs. (6)–(13) can be obtained by simply exchanging *ψ* with 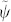. Hence, there is a gauge invariant quantity 
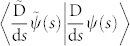
 which can be used to define a metric on ray space, 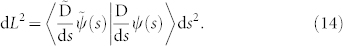
The metric Eq. (14) then determines the geodesic in ray space by variation of the length 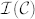


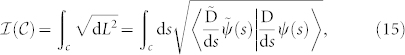
from which one can obtain a pair of geodesic equations, 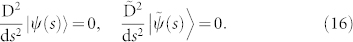
Eq. (16) are gauge covariant under local gauge transformation Eq. (4) and must hold simultaneously.

### Theorem

*Let the two non-biorthogonal states* |ψ_1_〉, |ψ_2_〉 *be connected by any geodesic G^1,2^* *satisfying Eq. (16), then the generalized Pancharatnam's phase* 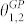 *shown in Eq. (7) is given by*

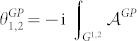
*where* 
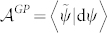
* is the local generalized Pancharatnam connection 1-form.*

*Proof*. Consider a geodesic |*φ*(*s*)〉 starting from |*φ*(0)〉 = |*ψ*_1_〉 and ending in the ray 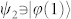 satisfying 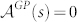, then geodesic equation Eq. (16) reduces to 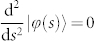, whose solution is a straight line described by 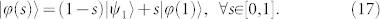
Let 
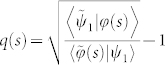
. It can be verified that *q*(0) = 0 and 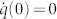 due to 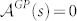 on the geodesic |*φ*(*s*)〉.

By inserting Eq. (17) into 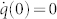, one can obtain 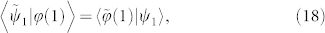
where we have used the binormalization condition 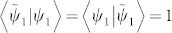. By inserting Eqs. (17) and (18) into *q*(*s*), then we have *q*(*s*) = 0, µ*s* ∈ [0, 1], which means |*ψ*_1_〉 and |*φ*(*s*)〉 are “in phase”. Due to the gauge covariance of the geodesic equation Eq. (16), let |*ψ*(*s*)〉 = e^i*θ*(*s*)^|*φ*(*s*)〉, µ*s* ∈ [0, 1], with the boundary condition *θ*(0) = 0 and 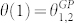, then |*ψ*(*s*)〉 is still a geodesic linking |*ψ*_1_〉 to |*ψ*_2_〉, which is denoted by *G*^1,2^.

And finally, 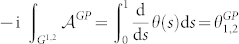. The proof is completed. 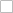


The theorem not only shows that a global generalized Pancharatnam's phase can be related with a local generalized Pancharatnam connection, but also indicates that the global generalized Pancharatnam's phase difference between the initial and final states is gauge-dependent and can not be considered as the geometric phase or Garrison-Wright's phase in a biorthogonal quantum system. Nevertheless, we have obtained a fundamental theorem in which the expression of the generalized Pancharatnam's phase presented is the main result of the paper. In the following sections, we will discuss how to apply the theorem to construct a universial theoretical framework for extending and unifying the family of Garrison-Wright's phases.

## The Family of Garrison-Wright's Phases: A Unifying Scheme

To extend and unify the family of Garrison-Wright's phases, we here repeatedly apply the theorem presented in previous section to the *N* vertices 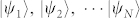, which satisfy 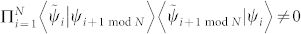 and can be linked one-by-one by *N* − 1 geodesics 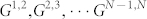 to obtain the accumulated generalized Pancharatnam's phase 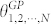 along the continuous curve 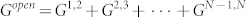 by 
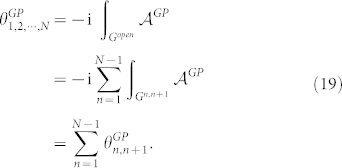
It should be noted that 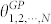 is not gauge invariant under local gauge transformation Eq. (4). However, if |*ψ_N_*〉 is linked back to |*ψ*_1_〉 by a geodesic *G^N^*^,1^ such that the curve 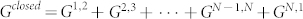 is continuous and closed, one can check that 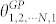 is gauge invariant and purely geometrical, 
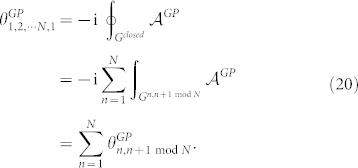
It should be noted that 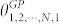 represents the geometric phase difference from |*ψ_N_*〉 to |*ψ*_1_〉. Generally, one may set |*ψ*_1_〉 and |*ψ_N_*〉 as the initial and the final states, respectively. Therefore, a universal formula for geometric phase between the initial |*ψ*_1_〉 and the final state |*ψ_N_*〉 is given by 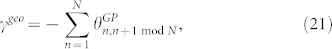
where the index *n* denotes the *n*-th vertice. The geometric phase as a gauge invariant in a biorthogonal quantum system merely depends on the path of the evolving state, and thus can resist to the noise not only brought by dynamics from system but also induced by decoherence from environment, because when the decoherence effect has been properly modeled into a quantum system, *e.g.*, Bethe-Lamb model for a metastable atomic system^33^, the corresponding geometric phase can additionally accommodate the information from decoherence as Garrison and Wright had shown in Ref. 7. Besides, once an appropriate model for a certain biorthogonal quantum system is built, Garrison-Wright's geometric phases and gauge potentials in such a system might be applied in the field of quantum simulation with neutral atoms^34^. Therefore, it is necessary to build a more general theory for the family of Garrison-Wright's phases. As a main result of the paper, the universal formula Eq. (21) will be used for the extension and unification of the family of Garrison-Wright's phases as follows, where not only the well-known Garrison-Wright's adiabatic and nonadiabatic geometric phases will be taken as two special examples in our theoretical framework, but Garrison-Wright's nonbiorthogonal and biorthogonal geometric phases will be introduced into the family.

### Adiabatic case

Consider a biorthogonal quantum system in an eigenstate |*n*(*R*_0_)〉 of hamiltonian *H*(*R*_0_), being adiabaticly transported round a circuit 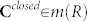 in the parameter manifold 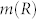 such that |*n*(*R_T_*)〉 = |*n*(*R*_0_)〉 after sufficiently long period of time *T*. Then, there exists an adiabatic geometric phase *γ^a^* given by Eq. (21) with the circuit **C***^closed^* being cut into sufficently many pieces, 
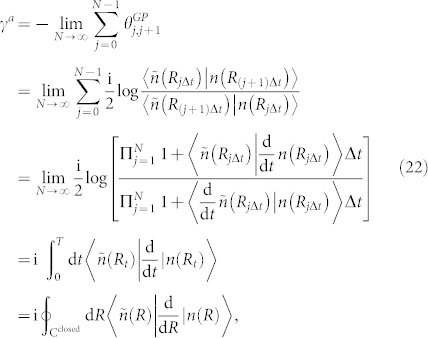
which exhibits the same result in adiabatic case of Garrison-Wright's work^7^. It should be noted that |*ψ*(*s*)〉 and its neighbors |*ψ*(*s* + d*s*)〉 can not be biorthogonal. Because the function |*ψ*(*s*)〉 and the parameter *s* are both continuous, however if 
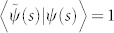
 but 
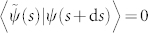
, the continuity will not hold, and here meets the contradiction.

### Nonadiabtic case

For any cyclic evolution of a biorthogonal quantum system, that is, the initial state |*ψ*_0_〉 is equivalent to the final state |*ψ_T_*〉 up to a phase factor e^i*ζ*^ as shown in Eq. (4), there exists a closed circuit 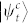 such that 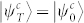. Then there exists a nonadiabatic geometric phase *γ^na^* as shown in the adiabatic case, 
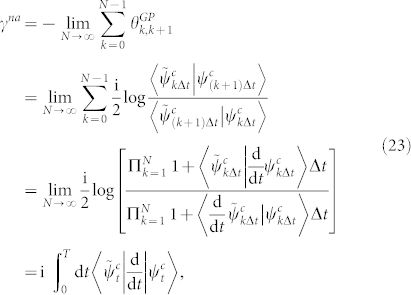
which is exactly the same to the result in nonadiabatic case of Garrison-Wright's work^7^. Besides, analogous to Pati's work^8^, another proposition also shows a way for nonadiabatic geometric phase and is given below.

### Proposition

*For an arbitrary cyclic evolution of a biorthogonal quantum system, the nonadiabatic geometric phase γ^na^ is manifested by the integral as*

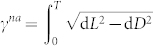
*where *
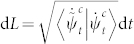
* is defined as an infinitesimal length of a closed circuit *
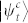
*, and *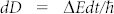 *is the infinitesimal distance between two neighboring rays with *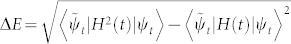 *representing the timedependent energy variance.*

*Proof*. According to Ref. 14, d*D* can be evaluated by 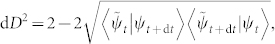
by Taylor expanding |*ψ_t_*_+d*t*_〉 and 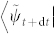 up to second order in time *t*, we have 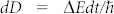 with 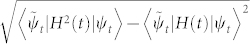. By calculating d*L*^2^ with the equivalence relationship 
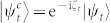
 and 
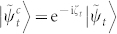
, 
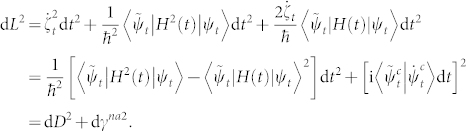


Here we have used 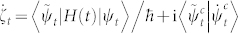. Finally, we integrate 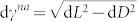 on the interval [0, *T*], and the proof is completed. 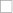


It should be noted that the proposition can be applied not only in calculating the nonadiabatic Garrison-Wright's phase but also in evaluating quantum speed limit (QSL)^35^ from the viewpoint of geometry.

### Nonbiorthogonal case

The evolving state |*ψ*(*t*)〉 of a biorthogonal quantum system governed by Schrödinger-like equation starts from an initial state |*ψ*(0)〉 and ends in the non-biorthogonal final state |*ψ*(*t*)〉, then the geometric phase *γ^nc^*(0, *t*) between |*ψ*(0)〉 and |*ψ*(*t*)〉 is given by Eq. (21) 
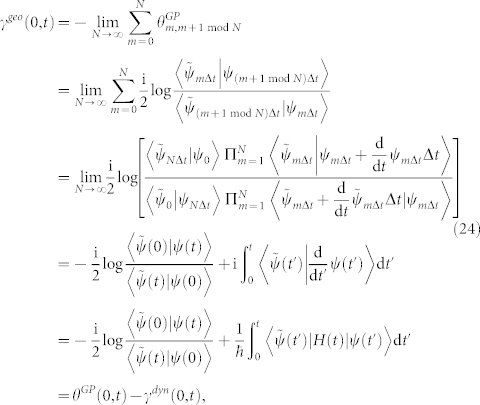
which coincides with the result of Ref. 14. It should be noted in Eq. (24) that the existence of *γ^geo^*(0, *t*) as well as *θ^GP^*(0, *t*) merely depends on whether the initial state is biorthogonal to the final state rather than any other intermediately traveled states, while the dynamical phase *γ^dyn^*(0, *t*) continuously exists.

### Biorthogonal case

As an extreme case of noncyclic evolution, geometric phase between biorthogonal states can not be evaluated directly by Eq. (21) or Eq. (24). However, if an intermediately traveled state |*ψ*(*t*_1_)〉 is non-biorthogonal to both the initial and the final states, then the geometric phase *γ^geo^*(0, *t*_1_, *t*) between the initial state |*ψ*(0)〉 and the final state |*ψ*(*t*)〉 can still be calculated indirectly by Eq. (24), 
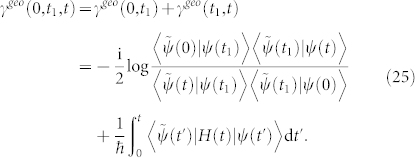
Here, the intermediately traveled state |*ψ*(*t*_1_)〉 acts as a torchbearer to guarantee that the geometric phase difference can be preserved and delivered from the initial state to the final biorthogonal state. Besides, |*ψ*(*t*_1_)〉 does not interrupt the process of the state evolution. Seen from another perspective, both the initial and the final states are projected onto the intermediately traveled state, and the total geometric phase difference is equal to the difference between *γ^geo^*(0, *t*_1_) and *γ^geo^*(*t*, *t*_1_), 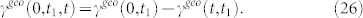
Hence, the intermediately traveled state |*ψ*(*t*_1_)〉 is unnecessary because it can be replaced with any state |*a*〉 which is non-biorthogonal to both the initial and final states to implement Eq. (26), 
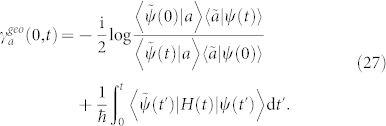
The first term on the right-hand side of Eq. (27) can be obtained by modifying Eq. (6), 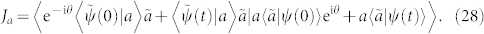
The second term on the right-hand side of Eq. (27) is to remove the dynamical phase off the final state |*ψ*(*t*)〉. However, neither Eq.(25) nor Eq.(27) determines a unique geometric phase between biorthogonal states due to many different choices of the intermediate state, such that 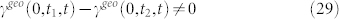
and 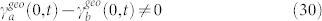


### Proposition

*There is a well-defined geometric phase of biorthogonal states, which is independent of intermidiate states.*

*Proof.* Two eigenstates |*j*(0)〉 and |*k*(0)〉 of *H*(*t*) evolve adiabatically to |*j*(*t*)〉 and |*k*(*t*)〉, respectively, such that 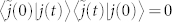 and 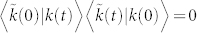. One can find an arbitrary intermediate state |*a*〉 which is not biorthogonal to |*j*(0)〉, |*j*(*t*)〉, |*k*(0)〉, and |*k*(*t*)〉. Then the geometric phase 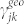 is given by 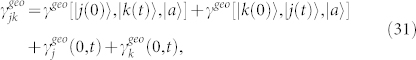
where 
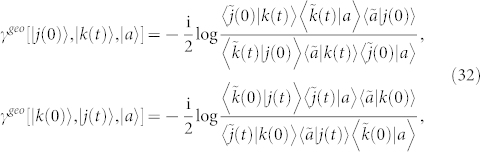
and 
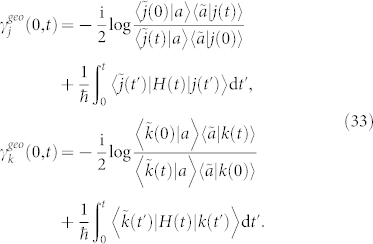
By some algebra, 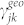 is independent on the choice of the intermediate state |*a*〉, *i.e.*, 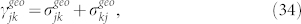
where 
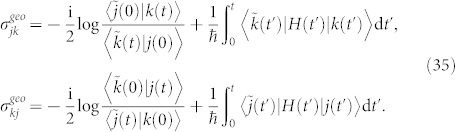
For more than two biorthogonal states, the similar procedure can be performed and gives, 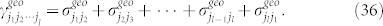
where *l* = 2, 3, …, *N*. The proof is completed.

The well-defined geometric phase of biorthogonal states is here called generalized Manini-Pistolesi's off-diagonal geometric phase, which is now introduced as a new member of the family of Garrison-Wright's phases. In the next section, we will exhibit how to apply our theory to a practical and physical issue of Garrison-Wright's phase.

## Applications

In the context of the two-level system with Weisskopf-Wigner decay included, Lamb *et al.* employed the Bethe-Lamb model to solve the problem that the matter-field interaction was sensitive to the choice of gauge when decaying state were used^33^. And then, Garrison and Wright also applied the model for exhibiting the complex-valued adiabatic and nonadibatic cyclic geometric phases^7^. Here we analyze the Bethe-Lamb model to illustrate applying our theory for exhibiting Garrison-Wright's phase which will no longer be confined to the adiabatic and nonadibatic cyclic cases.

The Bethe-Lamb hamiltonian for a two-level system with radiation damping, under the rotating wave approximation, is 
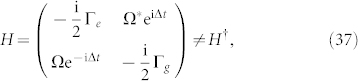
where Γ*_e_* and Γ*_g_* are the decay rates for excited and ground states, respectively; Δ = *ω*_0_ − *ω_L_* is the laser detuning frequency, and Ω is the Rabi frequency. By employing Floquet theorem^36^, one can obtain the explicit form for the nonunitary evolution operator which has been firstly given by Choutri *et al.*^37^. The evolution operators *U* for *H* and 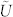 for *H*^†^ can be decomposed into three parts, respectively, 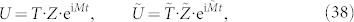
where 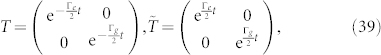

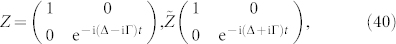

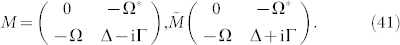
The corresponding eigenvalues are 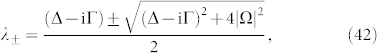

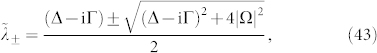
and eigenvectors are
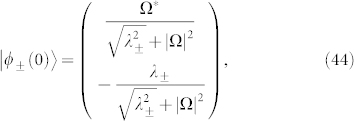

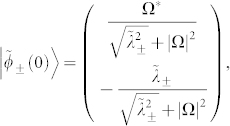
where 
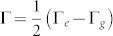
.

The ground state |*g*〉 = (0, 1)*^T^* is experimentally easy to prepare; however, because |*g*〉 is not a cyclic state, Garrison-Wright's phase initiating from it can not be evaluated by Garrison-Wright's formula^7^. For satisfying nondegenerate condition, Γ ≠ 4|Ω|^2^ is sufficient. By Eq. (24) and after some algebraic operations, one can obtain the noncyclic Garrison-Wright's phase initiating from |*g*〉 with an explicit expression. 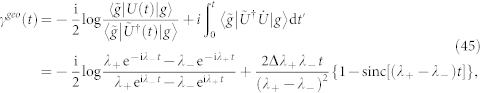
where 
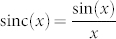
 is cardinal sine function, 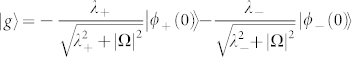 and 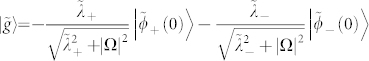, which satisfy initial condition 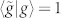.

As the laser detuning Δ = 0, the corresponding *γ^geo^*(*t*) is reduced to a simpler form, namely, 
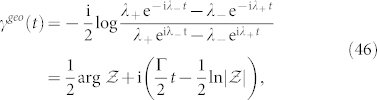
where 
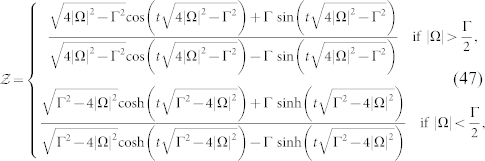
and complex logarithm function log(*z*) = ln|*z*| + i arg *z* has been used. Because 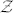 is always a real-valued number, the real part of *γ^geo^*(*t*) is to exhibit the sign change of 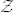 by Re*γ^geo^*(*t*) = 0 describing the positive sign of 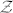 and Re*γ^geo^*(*t*) = *π*/2 describing the negative sign of 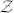, while the imaginary part of *γ^geo^*(*t*) is to meet their singular points. Moreover, the time of the sign change can be evaluated as follows: as 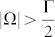, the first times for (+ → −) and (− → +) are 
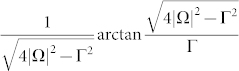
 and 
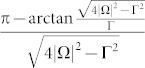
, respectively, and the period time is 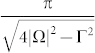; however, as 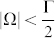, merely one time for (+ → −) is 
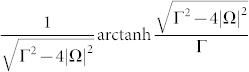
, and there is no time for (− → +).

**Figure 1:**
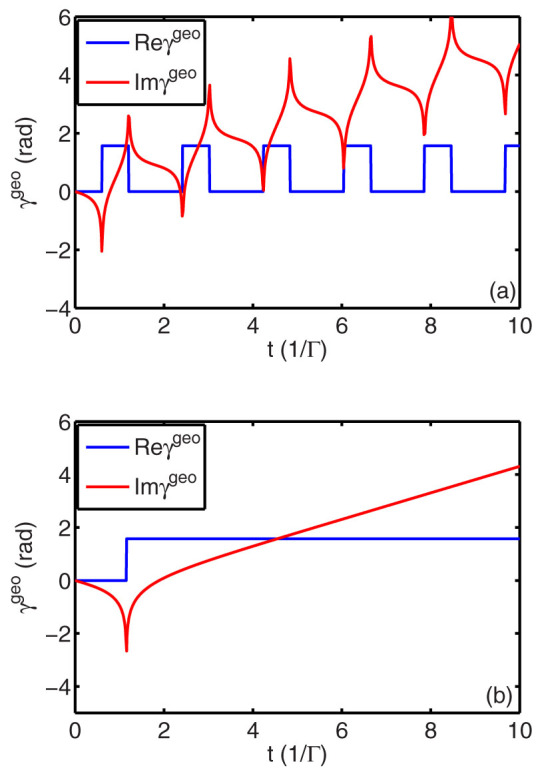
Garrison-Wright's phase initiating from ground state |*g*〉 varies over time when laser detuning Δ = 0. Other parameters are set as follows: for panel (a), Γ = 1, |Ω| = Γ; and for panel (b), Γ = 1, |Ω| = 0.4Γ.

We set Γ = 1 as the reference value for the other parameters in the Bethe-Lamb model. The modulo of Rabi frequency |Ω| can be modulated by varying the laser intensity. Without loss of generality, we choose |Ω| = Γ for the case 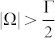 and |Ω| = 0.4Γ for the case 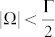. As illustrated in Fig. 1 (a), the real part of the Garrison-Wright's phase Re*γ^geo^*(*t*) shows a 1.8138/Γ-periodic square wave with the first rising edge at 0.6046/Γ and the first falling edge at 1.2092/Γ while the imaginary part of Im*γ^geo^*(*t*) exhibits singular points at rising and falling edges. Therefore, as |Ω| > Γ/2, Re*γ^geo^* can be applied to be a generator for periodic square waves whose waveforms can be modulated by Rabi frequency Ω for some fixed Γ, while Im*γ^geo^* can be applied to be another method to sharpen the rising and falling edges because its singular points can infinitely dilate and contract the modulo of the evolving wavefunction. As demonstrated in Fig. 1 (b), the real part of the Garrison-Wright's phase Re*γ^geo^*(*t*) has merely one rising edge at 1.1552/Γ but no falling edge while the imaginary part of Im*γ^geo^*(*t*) also exhibits singular points at the rising edge. Therefore, as |Ω| < Γ/2, Re*γ^geo^* can be applied to emulate Heaviside step function, while Im*γ^geo^* can also sharpen the rising edges where its singularity happens. Certainly, neither the generator for periodic square waves nor the emulator for Heaviside step function can last for long because the signal of the evolving wavefunction will essentially decline gradually due to the existance of 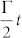 term in Im*γ^geo^*. However, it might be used in a considerable time scale for practical quantum computation and information^38^ because decoherence effect might be no longer a shortcoming but an advantage.

## Conclusion

In this paper, we have extended the concept of Pancharatnam's “in-phase” in interferometry based on generalized interference formula for biorthogonal quantum systems. Starting from the new concept, we have also introduced the corresponding parallel transport law, covariant derivative and geodesic equations which assist us to generalize Samuel-Bhandari's result to construct a unifying scheme for the family of Garrison-Wright's phases by generalizing Pancharatnam-Samuel-Bhandari's results. Not only have we exhibited how to integrate the known members of the family of Garrison-Wright's phases in the unifying scheme which has been verified to be useful in this paper, while a generalized Pati's result has also been introduced and might be applied in quantum speed limits, but also the new members, Garrison-Wright's nonbiorthogonal and biorthogonal geometric phases, have been introduced into the family. Especially, we have illustrated that the generalized Manini-Pistolesi's off-diagonal geometric phase for two and more biorthogonal states can still be well-defined, although the geometric phase between any two biorthogonal states are not uniquely defined. Finally, our thoery has been applied to provide an explicit result for Garrison-Wright's phase of a noncyclic ground state in Bethe-Lamb model, which implies a potential application in quantum computation and information.

## Author Contributions

X.C. and Y.Z. participated in the calculation of this work, wrote and revised this manuscript.

## Notes

Readers are welcome to comment on the online version of this article at www.nature.com/nature.
